# Functional proteins of mesenchymal stem cell-derived extracellular vesicles

**DOI:** 10.1186/s13287-019-1484-6

**Published:** 2019-11-28

**Authors:** Guanguan Qiu, Guoping Zheng, Menghua Ge, Jiangmei Wang, Ruoqiong Huang, Qiang Shu, Jianguo Xu

**Affiliations:** 1 Shaoxing Second Hospital, 123 Yanan Road, Shaoxing, 312000 Zhejiang China; 2 The Children’s Hospital of Zhejiang University School of Medicine, 3333 Binsheng Road, Hangzhou, 310051 Zhejiang China; 3 The First Affiliated Hospital of Zhejiang University School of Medicine, 79 Qingchun Road, Hangzhou, 310003 Zhejiang China

**Keywords:** Mesenchymal stem/stromal cells, Extracellular vesicles, Exosomes, Microvesicles, Functional proteins

## Abstract

Extracellular vesicles (EVs) contain proteins, microRNAs, mRNAs, long non-coding RNAs, and phospholipids, and are a novel mechanism of intercellular communication. It has been proposed that the immunomodulatory and regenerative effects of mesenchymal stem/stromal cells (MSCs) are mainly mediated by soluble paracrine factors and MSC-derived EVs (MSC-EVs). Recent studies suggest that MSC-EVs may serve as a novel and cell-free alternative to whole-cell therapies. The focus of this review is to discuss the functional proteins which facilitate the effects of MSC-EVs. The first section of the review discusses the general functions of EV proteins. Next, we describe the proteomics of MSC-EVs as compared with their parental cells. Then, the review presents the current knowledge that protein contents of MSC-EVs play an essential role in immunomodulation and treatment of various diseases. In summary, functional protein components are at least partially responsible for disease-modulating capacity of MSC-EVs.

## Background

Mesenchymal stem/stromal cells (MSCs) are obtainable from various human tissues and have multipotent differentiation aptitude. Due to their angiogenic, anti-apoptotic, and immunomodulatory capacities, MSCs may serve as a good candidate for regenerative medicine. Many studies including the work from our group [1] have examined the potential applications of MSCs in a variety of diseases including cardiomyopathy, kidney injuries, liver injuries, lung injuries, and cancers [2, 3]. Recent studies have demonstrated that the therapeutic benefits of MSCs are dependent on their extracellular vesicles (EVs).

According to the position statement of the International Society for Extracellular Vesicles (ISEV), EVs are particles, with a lipid bilayer, that are naturally released from the cells and cannot replicate [4]. The ISEVs have also stated that two categories (categories 1 and 2) of EV markers and one category of non-EV markers (category 3) must be examined to demonstrate the presence of EVs. Category 1 includes transmembrane or glycosylphosphatidylinositol-anchored proteins, indicating the lipid bilayer structure of EVs. Category 2 consists of cytosolic proteins such as enzymes, cytoskeletal proteins, and proteins actively incorporated into EVs via membrane binding. On the other hand, category 3 is composed of proteins of non-EV structures co-isolated with EVs, an indicator of impurity of the EV preparation [4]. EVs are typically classified into three subtypes according to sizes and biogenesis mechanisms: exosomes (50–150 nm), microvesicles (100–1000 nm), and apoptotic bodies (500–5000 nm). Exosomes are generated by several steps; endosomes formed from the plasma membrane, formation of intraluminal vesicles inside of multivesicular bodies (MVB) by inward budding, fusion of MVB with the plasma membrane, and secretion of internal vesicles. On the other hand, both microvesicles and apoptotic bodies are larger in size than exosomes and formed by local deformation and direct outward budding from the plasma membrane [5]. In cancer research, oncosomes, which function to transfer oncogenic signals across cell boundaries, refer to microvesicles or even all EVs that are released from cancer cells [6]. Due to overlapping size, density, and membrane composition, most of the current methods of EV isolation yield a mixed EV population [7]. There is still no consensus on specific markers of EV subtypes. Flotillin, heat-shock 70 kDa proteins, and major histocompatibility complex class I and class II are common markers for all types of EVs, while CD9, CD63, and CD81 tetraspanins are generally classified as exosome markers [8].

Almost all organisms and cells are capable of producing EVs. MSC-derived EVs (MSC-EVs) bear not only the markers that are characteristic of MSCs such as CD29, CD73, CD90, CD44, and CD105, but also EV markers such as CD63, CD9, and CD81 [9]. To apply MSC-EVs in clinical studies, implementation of a good manufacturing practice-compliant protocol is necessary. To scale up the production of MSC-EVs, 3D bioreactors may be required to provide a homogeneous distribution of nutrients and oxygen while monitoring cell number, viability, and proliferation [10]. Xenogenic components such as fetal bovine serum should be substituted with human serum or human platelet lysate. For example, platelet lysate has been used to generate MSC-EVs for treating refractory graft-versus-host disease [11]. Differential ultracentrifugation has been the most widely used method for EV separation. However, ultracentrifugation has several drawbacks, including low purity and induced aggregation of EVs, and thus is not suitable for large-scale purification of MSC-EVs [12]. Instead, MSC-EVs purified via precipitation with polyethylene glycol have been applied for clinical investigation [11]. It has been proposed that the ratio between particle number and protein concentration may serve as an indicator for purity in EV production [13].

MSC-EVs exert their functions via the transfer of their contents, such as proteins, mRNAs, and microRNAs (miRNAs), to target cells. There was a horizontal transfer of insulin-like growth factor-1 receptor mRNA from EVs to cisplatin-damaged proximal tubular cells, which increased the proliferation of proximal tubular epithelial cells [14]. In mice, MSC-EVs alleviated lipopolysaccharide-induced acute lung injury via their content of angiopoietin-1 mRNA [15]. MSC-EV-mediated transfer of miRNAs is an area of active research. Transfer of miRNAs from MSC-EVs to target cells has been demonstrated as the underlying mechanisms in alleviating injury of the kidney [16], heart [17], liver [18], and brain [19]. The present review article will cover the general functions of EV proteins and proteomics of MSC-EVs and emphasize on functional proteins mediating the effects of MSC-EVs in different diseases.

## General functions of EV proteins

EV proteins participate in EV biogenesis, sort EV cargo, control EV release, and facilitate interactions between EVs and recipient cells. EV biogenesis can be categorized into endosomal sorting complex required for transport (ESCRT)-dependent and ESCRT-independent pathways. ESCRT consists of 4 protein complexes, ESCRT-0, I, II, and III, with over 20 ESCRT and accessory proteins such as CHMP4C, VPS4B, VTA1, and ALIX. In HeLa-CIITA cells, silencing of ESCRT-0 subunit Hrs, ESCRT-I protein STAM1, or ESCRT-I protein TSG101 altered the size and/or protein contents of secreted EVs. The depletion of VPS4B did not affect the size and composition of EVs while elevated the secretion [20]. In head and neck squamous cell carcinoma cells, knockdown of ESCRT-0 subunit Hrs reduced the secretion of EVs per cell [21]. Biogenesis of EVs can also arise from the ESCRT-independent pathway. In human epithelial type 2 cells, depletion of key subunits of all 4 ESCRT inhibited the formation of EGF-induced EVs, but EGF-independent EVs remained intact [22]. On the other hand, tetraspanins such as CD63, CD53, CD37, CD81, and CD9, which are among the most enriched membrane proteins in EVs, play an essential role in the ESCRT-independent pathway. The release of EVs was defective from dendritic cells generated from CD9 knockout mice compared with wild type. In addition, less amount of flotillin-1 was observed in EVs from dendritic cells of the knockout mice [23]. Hurwitz et al. found that secretion of small EVs was significantly decreased in HEK293 cells with CD63 gene knockout, indicating the role of CD63 in the biogenesis of EVs [24]. The same group also reported that latent membrane protein 1, an Epstein-Barr virus-encoded oncoprotein, accumulated in CD63-positive EVs. Levels of latent membrane protein 1 were reduced after CD63 knockout in nasopharyngeal carcinoma cells [25].

Sorting of EV cargo is also regulated by both ESCRT-dependent and ESCRT-independent pathways. The three early-acting ESCRT complexes (ESCRT-0, ESCRT-I, and ESCRT-II) harbored ubiquitin-binding domains and could interact with ubiquitinated cargo, sorting ubiquitinated proteins into EVs [26]. Proteins lacking ubiquitin modification could still be sorted into EVs in an ESCRT-dependent pathway. ESCRT-associated protein ALIX bound with protease-activated receptor-1 and the purinergic receptor P2Y_1_ via a YPX_3_L motif [27, 28]. ESCRT-0 subunit Hrs interacted directly with interleukin-2 receptor β [29]. However, the downstream ESCRT-III and the Vps4 complex were required for the sorting of ubiquitin lacking proteins [28]. On the other hand, tetraspanin CD63 played a direct role in ESCRT-independent sorting of protein and EV formation in melanocytes [30]. Sorting of Argonaute 2, which is a critical RNA-induced silencing complex (RISC) component and a regulator for miRNA sorting, was controlled by KRAS-MEK signaling in colon cancer cell lines [31]. Sortilin, a sorting protein that directs target proteins to EVs, regulated its own trafficking to EVs via dimerization in HEK293 cells [32].

It is well known that EV fusion and release are facilitated by soluble *N*-ethylmaleimide-sensitive factor attachment protein receptors (SNAREs) [33]. EV release is also controlled by EV proteins such as Rab GTPases, which serve as essential regulators of membrane trafficking. The release of EVs was slightly elevated in K562 cells transfected with plasmid DNA encoding for wild-type Rab11, while overexpression of a dominant-negative Rab11 mutant blocked EV secretion [34]. Rab7 and Rab27b controlled the secretion of EVs containing miR-143 from endothelial cells induced by KLF2 [35]. Hsu et al. showed that Rab35 regulated the release of proteolipid protein in EVs of oligodendroglial cells by adjusting the docking/tethering of MVB with the plasma membrane [36]. Rab27a and Rab27b played different roles in the secretion of EVs. Rab27a silencing significantly increased the size of MVB, while the distribution of MVB was around the perinuclear region after Rab27b silencing in HeLa cells [37].

The interactions between EVs and recipient cells involve surface proteins of EVs such as integrins, tetraspanins, and ICAMs, which interact with the target cells via receptor-ligand binding. When intravenously injected, integrin α_6_β_4_- and α_6_β_1_-enriched EVs from tumors co-localized with fibroblasts and epithelial cells in laminin-rich lung microenvironments. Integrin α_v_β_5_-expressing EVs from tumors fused with Kupffer cells in the fibronectin-rich liver. Meanwhile, integrin β_3_-abundant EVs interacted primarily with CD31+ brain endothelial cells [38]. EVs containing tetraspanin8 and its associated CD49d from an adenocarcinoma model were internalized by endothelial cells via CD106, a ligand for CD49d. Uptake of tetraspanin8-CD49d complex-containing EVs initiated angiogenesis-related gene expression in endothelial progenitors [39]. ICAM-1 is a ligand for the lymphocyte function-associated antigen 1. ICAM-1 from mature dendritic cell-derived EVs was essential for effective naive T cell priming [40]. Other EV proteins also regulate the uptake of EVs by target cells. In pancreatic ductal adenocarcinoma, the healthy pancreatic tissue surrounding the tumor released REG3β. REG3β bound to the glycoproteins on the surface of circulating EVs, resulting in reduced internalization of EVs by tumor cells [41].

## Proteomics of MSC-EVs

An increasing number of studies have reported the proteomic signature of MSC-EVs. A summary of the data is presented in Table 1. Kim et al. identified 730 proteins in the proteomic analysis of human bone marrow (BM)-derived MSC-EVs. The MSC-EV proteins contained markers of MSCs and EVs as well as signaling molecules regulating the self-renewal and differentiation capacities of MSCs. In addition, the list included proteins participating in cell proliferation, adhesion, migration, and morphogenesis [42]. In another study of human BM-derived MSC-EVs, Angulski et al. identified 797 proteins, 60% of which overlapped with those of Kim et al. [43]. Anderson et al. compared the proteomic profile of EVs from BM-derived MSCs under normoxic and hypoxic conditions and identified a total of 1927 proteins, 457 of which were not found in MSCs, suggesting protein enrichment in MSC-EVs. Hypoxic treatment promoted the expression of angiogenic proteins including platelet-derived growth factor, fibroblast growth factor, and epidermal growth factor [44]. La Greca et al. examined the proteomics of pluripotent stem cell-derived MSCs as well as their EVs and identified 1483 and 560 unique proteins, respectively. In addition, EVs shared 37.32% of the proteins with parental cells and enriched with immune, extracellular matrix, and cell adhesion molecules [45]. By combining the results of 3 sets of proteomics of EVs from human embryonic stem cell-derived MSCs, Lai et al. detected 766 proteins in mass spectrometry analysis. By antibody array, they discovered 101 proteins with 10 proteins overlapping with mass spectrometry analysis. Functional analysis revealed these 857 proteins from MSC-EVs were associated with biological processes such as communication, motility, inflammation, and biogenesis of EVs [46]. They later divided EVs from human embryonic stem cell-derived MSCs into 3 types based on their affinities for membrane lipid-binding ligands: cholera toxin B chain (CTB), annexin-V (AV), or Shiga toxin (ST). All CTB-, AV-, and ST-binding EVs contained actin. EV binding activity of CTB and AV was mutually exclusive, while fibronectin was only present in ST-EVs, indicating that these 3 EV types are unique entities. The plasma membrane and cytoplasm of MSCs contained CTB- and AV-binding activity, while the nucleus had ST-binding activity, suggesting that the 3 EV types have different biogenesis and serve different biological functions. They also determined the proteomics of the CTB-binging EVs and CTB-depleted fractions. CTB-binding fraction and CTB-depleted fraction had 1806 and 1547 proteins, respectively, with 987 proteins in both fractions. Proteins in CTB-binging EVs were associated with the endosomal biogenesis of EVs [47]. Otero-Ortega et al. studied the application of rat adipose-derived MSC-EVs in an experimental animal model of subcortical stroke. Proteomic analysis of the MSC-EVs identified 2416 proteins. The majority of these proteins were associated with brain repair function and had hydrolase activity, tubulin binding ability, and kinase activity [48]. Eirin et al. reported the finding of 5469 proteins in adipose-derived pig MSCs and 4937 in MSC-EVs. In differential expression analysis, 128 proteins were enriched in MSC-EVs versus MSCs, while 563 proteins were absent in EVs. The enriched proteins were associated with MSC-mediated tissue regeneration such as angiogenesis, coagulation, apoptosis, inflammation, and extracellular matrix remodeling. The absent proteins were mostly nuclear proteins [49]. In an integrated transcriptomic and proteomic analysis of MSC-EVs derived from pig adipose-derived MSCs, Eirin et al. found 5623 EV proteins with 4 miRNAs, 255 mRNAs, and 277 proteins enriched in EVs versus MSCs. EV-enriched miRNAs and mRNAs were related to transcription factors, while EV-enriched proteins participated in extracellular matrix, coagulation, inflammation, and angiogenesis [50]. Recently, the same group performed a study comparing the proteomics of adipose-derived MSC-EVs to their parent MSCs from pigs with metabolic syndrome and normal control. The proteomic analysis discovered 6690 and 6790 distinct proteins in MSC-EVs from control and metabolic syndrome, respectively. In normal control, 146 proteins were enriched in MSC-EVs versus MSCs, whereas 787 proteins were enriched in MSC-EVs versus MSCs from pigs with metabolic syndrome. Proteins participating in vesicle transport and cell-to-cell communication were enriched in both types of MSC-EVs. Proteins upregulated only in control MSC-EVs were associated with tissue regeneration. On the other hand, proteins upregulated only in MSC-EVs from pigs with metabolic syndrome were connected with pro-inflammatory pathways [51]. By analyzing datasets of published MSC-EV proteomics, an MSC-EV-specific protein signature was reported with 22 members and involved in functions such as cell adhesion and integrin-receptor interaction [52].

**Table 1:** Proteomic studies of MSC-EVs

References	Sources of MSCs and MSC-EVs	Analytic techniques	Major findings
Kim et al. [42]	Human BM-derived MSC-EVs	LC-MS/MS	730 proteins including markers of MSCs and EVs as well as proteins involved in the therapeutic effects of MSCs
Angulski et al. [43]	Human BM-derived MSC-EVs	Nano LC-MS/MS	797 proteins with 60% overlapping with those of Kim et al.
Anderson et al. [44]	Human BM-derived MSC-EVs	Nano LC-MS/MS	Total 1927 proteins under normoxic and hypoxic conditions, with increased expression of angiogenic proteins under hypoxic condition
La Greca et al. [45]	Human pluripotent stem cell-derived MSC-EVs	LC-MS/MS	560 proteins enriched with immune, extracellular matrix, and cell adhesion molecules compared with MSCs
Lai et al. [46]	Human embryonic stem cell-derived MSC-EVs	LC-MS/MS and antibody arrays	766 proteins via MS analysis and 101 via antibody array, with functions such as communication, motility, inflammation, and biogenesis of EVs
Lai et al. 2016 [47]	Human embryonic stem cell-derived MSC-EVs	LC MS/MS	1806 proteins in CTB-bound fraction, 1547 proteins in CTB-depleted fraction, and 987 proteins in both fractions, with function of EV biogenesis in CTB-bound fraction
Otero-Ortega et al. [48]	Rat adipose-derived MSC-EVs	LC-MS/MS	2416 proteins, a big percentage of which was related to brain repair function
Eirin et al. [49]	Pig adipose-derived MSC-EVs	LC-MS/MS	4937 proteins with 128 enriched proteins, which were associated with tissue regeneration
Eirin et al. [50]	Pig adipose-derived MSC-EVs	LC-MS/MS	5623 proteins with 277 enriched proteins along with 4 enriched miRNAs and 255 enriched mRNAs in an integrated transcriptomic and proteomic analysis
Eirin et al. [51]	Adipose-derived MSC-EVs from pigs with metabolic syndrome or control	LC-MS/MS	6690 proteins with 146 enriched proteins relating regeneration in control EVs. 6790 proteins with 787 enriched proteins relating pro-inflammatory pathways in EVs from metabolic syndrome

*MSC-EVs* mesenchymal stem cell-derived extracellular vesicles, *BM* bone marrow, *LC-MS/MS* liquid chromatography with tandem mass spectrometry, *CTB* cholera toxin B chain

## Functional proteins of MSC-EVs in immunomodulation

Like their parental cells, MSC-EVs have also been investigated for their potent immunomodulatory capacity. A list of functional proteins of MSC-EVs involved in immunoregulation and pathophysiology of human diseases is summarized in Table 2. Accumulating evidence has demonstrated that MSC-EVs modulate innate and adaptive immune responses through interaction with immune effector cells such as T, B, natural killer, and dendritic cells. Di Trapani et al. demonstrated that there was a direct correlation between EV uptake by immune effector cells and immunomodulation, suggesting the modulatory effects were transferable [79]. Several studies have evidenced that the immunomodulatory function of MSC-EVs could be, at least in part, facilitated by functional proteins. MSC-EVs from mouse BM were shown to produce tolerogenic molecules such as programmed death ligand-1 (PD-L1), galectin-1, and transforming growth factor β (TGF-β). MSC-EVs inhibited the proliferation of auto-reactive lymphocytes acquired from mice with experimental autoimmune encephalomyelitis. MSC-EVs also increased the secretion of anti-inflammatory cytokines such as IL-10 and TGF-β and promoted the generation of regulatory T cells [53]. MSC-EVs derived from dog Wharton’s jelly had a dose-dependent suppressive effect on CD4+ T cell proliferation in vitro, which was absent after EV depletion. MSC-EVs contained TGF-β, which was likely tethered to EVs by betaglycan. EV suppression of CD4+ T cell proliferation was blocked by an antagonist for TGF-β receptor 1 and neutralizing antibodies to TGF-β [54]. In a similar study, Alvarez et al. documented that human endometrial MSC-EVs inhibited CD4+ T cell activation in stimulated lymphocytes. MSC-EVs had high active TGF-β expression compared with EV free concentrated supernatants. TGF-β neutralizing antibodies blocked the immunomodulatory activity of MSC-EVs [55]. Adamo et al. found that EVs from both resting and inflammation-primed MSCs were internalized by activated B cells. Proteins with immunomodulatory potential such as LG3BP and PTX3 were upregulated, while MOES and S10A6 were downregulated in inflammation-primed MSC-EVs compared with resting EVs. Treatment of activated B cells with inflammation-primed MSC-EVs induced a significant down-modulation of the PI3K-AKT signaling pathway and modulated actin cytoskeleton during B cell spreading [56]. Harting et al. discovered that EVs from inflammation-stimulated MSCs reduced TNF-α and IFN-γ release from activated splenocytes compared with control EVs. Both control and stimulated MSC-EVs were internalized by peripheral blood mononuclear cells. Furthermore, stimulated MSC-EVs had an elevated expression of COX2, which participated in the TNF-α inhibition [57].

**Table 2:** Studies demonstrating the functional proteins in MSC-EVs

References	Sources of MSC-EVs	Experimental model	Functional protein	Major findings
Mokarizadeh et al. [53]	Mouse BM MSC-EVs	Mouse with experimental autoimmune encephalomyelitis	PD-L1, galectin-1, and TGF-β	MSC-EVs elevated the production of anti-inflammatory cytokines and the generation of regulatory T cells on splenic mononuclear cells.
Crain et al. [54]	Wharton’s jelly MSC-EVs of dog	In vitro peripheral blood mononuclear cells	TGF-β	MSC-EVs suppressed CD4+ T cell proliferation via TGF-β.
Alvarez et al. [55]	Human endometrial MSC-EVs	In vitro peripheral blood mononuclear cells	TGF-β	The immunomodulatory effect of MSC-EVs on CD4+ T cells is partially mediated by TGF-β.
Adamo et al. [56]	Human BM MSC-EVs	Culture of B cells with inflammation-primed MSC-EVs	MOES, LG3BP, PTX3, and S10A6	Inflammation-primed MSC-EVs modulated the PI3K-AKT signaling pathway of B cells and the actin cytoskeleton.
Harting et al. [57]	Human BM MSC-EVs	Culture of splenocytes with MSC-EVs	Cox2	EVs from inflammation-stimulated MSCs attenuated inflammation.
Chen et al. [58]	Human BM MSC-EVs	Mouse model for inducible hippocampal CA1 neuron damage	IL-2, IL-10, RANTES, VEGF, and BDNF	EP4 antagonist induced the release of MSC-EVs and improved memory and learning deficiencies.
Katsuda et al. [59]	Human adipose MSC-EVs	In vitro model of Alzheimer’s disease	Neprilysin	MSC-EVs carried enzymatically active neprilysin, which degrades β-amyloid peptide.
de Godoy et al. [60]	Rat BM MSC-EVs	In vitro model of Alzheimer’s disease	Catalase	MSC-EVs protected neurons from β-amyloid peptide-induced oxidative stress via the transfer of catalase.
McBride et al. [61]	Human BM MSC-EVs	Fibroblasts from a patient with epidermolysis bullosa	Type VII collagen	MSC-EVs transported type VII collagen protein and mRNA to fibroblasts.
Zhang et al. [62]	Human umbilical cord MSC-EVs	Rat skin wound model	Wnt4	MSC-EVs delivered Wnt4 to skin cells. Wnt4 enhanced wound healing and improved the survival of skin cells.
Zhang et al. [63]	Human umbilical cord MSC-EVs	Rat skin wound model	14-3-3ζ	MSC-EVs delivered 14-3-3ζ to keratinocytes and controlled the Wnt response via regulating YAP.
Shabbir et al. [64]	Human BM MSC-EVs	In vitro model of wound healing	STAT3	MSC-EVs enhanced the proliferation and migration of diabetic wound fibroblasts and augmented endothelial angiogenesis.
Ahn et al. [65]	Human umbilical cord blood MSC-EVs	Neonatal rat hyperoxic lung injury	VEGF	MSC-EVs attenuated neonatal hyperoxic lung injuries via the transfer of VEGF, and the effect was lost in MSC-EVs with VEGF knockdown.
Wang et al. [66]	Mouse BM MSC-EVs	In vitro LPS-induced endothelial cell injury	HGF	MSC-EVs stabilized endothelial barrier function via HGF, and the effect was blocked by knockdown of HGF.
Gennai et al. [67]	Human BM MSC-EVs	Ex vivo human lung perfusion model	CD44	MSC-EVs restored alveolar fluid clearance in donor human lung, and the effect was blocked by anti-CD44 antibody
Hu et al. 2018 [68]	Human BM MSC-EVs	Injured lung microvascular endothelial cells	CD44	MSC-EVs restored protein permeability in injured microvascular endothelial cells via CD44-mediated EV internalization.
Eirin et al. [69]	Pig adipose autologous MSC-EVs	Pig model of renal artery stenosis	IL-10	MSC-EVs attenuated renal inflammation and fibrosis, and the protection was blunted in IL-10-depleted MSC-EVs.
Shen et al. [70]	Mouse BM MSC-EVs	Mouse ischemia/reperfusion model	CCR2	MSC-EVs blocked macrophage functions and alleviated ischemia/reperfusion-induced renal injury via CCR2.
Jiang et al. [71]	Human urine MSC-EVs	In vitro and in vivo model of diabetic nephropathy	VEGF, TGF-β, and angiogenin	MSC-EVs averted kidney complications from type I diabetes in rats by suppressing apoptosis and promoting angiogenesis.
Ma et al. [72]	Human umbilical cord MSC-EVs	Rat model of acute myocardial infarction	PDGF-D	EVs derived from MSCs with AKT overexpression enhanced angiogenesis via PDGF-D.
Gonzalez-King et al. [73]	Human dental pulp MSC-EVs	In vitro and in vivo model of angiogenesis	Jagged1	HIF1-α overexpressing MSC-EVs promoted angiogenesis via elevated level of Jagged1.
Vrijsen et al. [74]	Mouse BM MSC-EVs	In vitro and in vivo model of angiogenesis	EMMPRIN	MSC-EVs stimulated angiogenesis via elevated expression of EMMPRIN.
Lopatina et al. [75]	Human adipose MSC-EVs	In vitro and in vivo model of angiogenesis	c-kit and SCF	EVs from PDGF-treated MSCs carried c-kit and SCF that displayed a role in angiogenesis.
Wysoczynski et al. [76]	Human cardiac MSC-EVs	In vitro model of angiogenesis	Angiopoietins 1 and 2	The pro-angiogenic effects of MSC-EVs were independent of RNA transfer and relied on packaged angiopoietins 1 and 2.
Iglesias et al. [77]	Human amniotic fluid MSC-EVs	In vitro model of cystinosis	Cystinosin	MSC-EVs alleviated cystine accumulation in skin fibroblasts from cystinosis patients via the transfer of cystinosin.
Mao et al. [78]	Mouse BM MSC-EVs	Mouse foregastric carcinoma in vitro and in vivo	UBR2	UBR2 was enriched in MSC-EVs with p53 deficiency and promoted gastric cancer progression via the Wnt/β-catenin pathway.

*BM* bone marrow, *MSC-EVs* mesenchymal stem cell-derived extracellular vesicles, *PD-L1* programmed death ligand-1, *TGF-β* transforming growth factor β, *EP4* prostaglandin E_2_ receptor 4, *BDNF* brain-derived neurotrophic factor, *VEGF* vascular endothelial growth factor, *HGF* hepatocyte growth factor, *PDGF-D* platelet-derived growth factor D, *HIF1-α* hypoxia-inducible factor 1-alpha, *EMMPRIN* extracellular matrix metalloproteinase inducer, *SCF* stem cell factor, *UBR2* ubiquitin protein ligase E3 component n-recognin 2

## Functional proteins of MSC-EVs in neurological diseases

Chen et al. reported that the treatment of MSCs with GW627368X, a prostaglandin E_2_ receptor 4 (EP4) antagonist, promoted the release of MSC-EVs. The GW627368X-induced MSC-EVs had elevated levels of anti-inflammatory cytokines and neuron-supporting proteins including IL-2, IL-10, RANTES, vascular endothelial growth factor (VEGF), and brain-derived neurotrophic factor (BDNF). In a mouse model for inducible hippocampal CA1 neuron damage, GW627368X-elicited MSC-EVs improved memory and learning deficiencies triggered by hippocampus damage. Furthermore, the induced MSC-EVs elevated the expression of genes implicated in astrocyte differentiation, blood-brain barrier, and anti-inflammation [58]. In an in vitro model of Alzheimer’s disease, MSC-EVs were transferred into neuroblastoma N2a cells and decreased both extracellular and intracellular β-amyloid peptide levels. In addition, MSC-EVs carried enzymatically active neprilysin, which is an essential molecule in degrading β-amyloid peptide in the brain [59]. In another in vitro model of Alzheimer’s disease, MSC-EVs protected neurons against β-amyloid peptide-induced oxidative stress and synapse damage similar to that of parental MSCs. MSC-EVs exhibited the neuroprotective effect via the delivery of catalase [60].

## Functional proteins of MSC-EVs in dermatological diseases

Results from clinical trials have shown that MSCs were effective in the treatment of epidermolysis bullosa, a genetic skin condition resulting from a lack of type VII collagen production, by improving the reconstruction of the basement membrane and healing of cutaneous wound [80]. In vitro, MSC-EVs transferred both type VII collagen protein and mRNA to fibroblasts from a patient with epidermolysis bullosa [61]. In a rat model of burn injury, Zhang et al. found that MSC-EVs delivered Wnt4 to skin cells, promoted wound healing via activation of Wnt/β-catenin signaling, and inhibited skin cell apoptosis via activation of the AKT pathway. The effects of MSC-EVs on skin cells were abolished by knockdown of Wnt4 expression [62]. The same group also demonstrated that MSC-EVs restricted excessive cell proliferation and expansion in the tissue remodeling phase via delivering of 14-3-3ζ to skin cells. Under high cell density conditions, 14-3-3ζ induced YAP phosphorylation, which inhibited Wnt/β-catenin signaling [63]. In an in vitro model of wound healing, Shabbir et al. demonstrated that MSC-EVs were taken up by fibroblasts and promoted the growth and migration of fibroblasts from normal and diabetic chronic wounds in a dose-dependent manner. MSC-EVs also enhanced the angiogenesis of human umbilical vein endothelial cells. Additionally, MSC-EVs were enriched with transcriptionally active STAT3, an inducer for growth factors [64].

## Functional proteins of MSC-EVs in pulmonary diseases

Ahn et al. demonstrated that MSC-EVs were as effective as MSCs in alleviating neonatal hyperoxic lung injury in a rat model. The protective effects were lost in MSC-EVs with VEGF-knockdown, indicating the protection was mediated by the transfer of VEGF [65]. In an in vitro model of LPS-induced endothelial cell injury, Wang et al. found that treatment with MSC-EVs reduced endothelial paracellular and transcellular permeabilities, and the effect was abolished after knockdown of hepatocyte growth factor (HGF) in MSCs. MSC-EVs also diminished endothelial apoptosis and elevated the expression of junction proteins VE-cadherin and occludin. In addition, treatment with MSC-EVs decreased IL-6 level and elevated IL-10 level in the media of endothelial cells via an HGF-dependent manner [66]. Using an ex vivo lung perfusion model, Gennai et al. discovered that MSC-EVs promoted alveolar fluid clearance, reduced lung weight gain, and improved lung compliance. Anti-CD44 antibody blocked the effects of MSC-EVs, indicating the role of CD44 in mediating internalization of the MSC-EVs into the injured cells in the lung [67]. Hu et al. revealed that the administration of MSC-MVs restored the permeability of injured human lung microvascular endothelial cells. MSC-EVs prevented F-actin reorganization and restored the function of junction proteins ZO-1 and VE-cadherin in injured endothelial cells. Pretreatment of MSC-EVs with an anti-CD44 antibody reduced the beneficial effect, indicating that the internalization of MSC-EVs into the endothelial cells is an essential process [68].

## Functional proteins of MSC-EVs in renal diseases

In a pig model of renal artery stenosis, treatment with MSC-EVs restored renal blood flow and alleviated renal inflammation and fibrosis. Fragments of EVs co-localized with kidney tubular cells and macrophages, suggesting internalization of EVs. The protective effects were abrogated in pigs treated with IL-10-depleted MSC-EVs [69]. In a mouse model of ischemia/reperfusion-induced renal injury, MSC-EVs suppressed macrophage function and attenuated renal injury. MSC-EVs were enriched with C-C motif chemokine receptor-2 (CCR2). Knockdown of CCR2 abolished the protective effect of MSC-EVs [70]. In a rat model of type I diabetes, Jiang et al. documented that human urine MSC-EVs alleviated diabetes-induced kidney injury by inhibiting the apoptosis of podocytes and tubular epithelial cells and increasing the proliferation of glomerular endothelial cells. MSC-EVs contained angiogenesis factors such as VEGF, TGF-β, and angiogenin [71].

## Functional proteins of MSC-EVs in cardiovascular diseases

In a rat model of acute myocardial infarction, EVs derived from MSCs with AKT overexpression improved cardiac function through the promotion of angiogenesis. The MSC-EVs with AKT overexpression displayed a higher level of platelet-derived growth factor D (PDGF-D) compared with control MSC-EVs. Transfection of PDGF-D-siRNA into MSCs abolished the angiogenesis effect of MSC-EV in endothelial cells [72]. Gonzalez-King et al. demonstrated that Jagged1 was more abundant in hypoxia-inducible factor 1-alpha (HIF1-α) overexpressing MSC-EVs than in EV control. HIF1-α overexpressing MSC-EVs enhanced angiogenesis as revealed in endothelial tube formation assay in vitro and Matrigel plug assay in vivo. In addition, HIF1-α-induced angiogenesis effects were abolished by prior incubation of MSC-EVs with an anti-Jagged 1 antibody [73]. Vrijsen et al. reported that MSC-EVs were efficiently taken up by endothelial cells, enhanced endothelial vessel formation in vitro, and induced angiogenesis in Matrigel plug assay in vivo. Elevated expression of extracellular matrix metalloproteinase inducer (EMMPRIN) was revealed in MSC-EVs. Pro-angiogenic effect was abolished by knockdown of EMMPRIN [74]. In addition, Lopatina et al. discovered that PDGF stimulated the release of EVs from adipose-derived MSCs. EVs derived from PDGF-treated MSCs had elevated expression of pro-angiogenic c-kit and stem cell factor (SCF) and enhanced angiogenesis in vitro and in vivo. Blockade of SCF or c-kit with specific antibodies abrogated the pro-angiogenic activity of the MSC-EVs [75]. Furthermore, Wysoczynski et al. found that human cardiac MSC-EVs promoted cell migration, tube formation, and survival of human umbilical vein endothelial cells in vitro. There was an enrichment of angiopoietins 1 and 2 in MSC-EVs as revealed by cytokine array analysis. Inhibition of Tie2, a receptor for angiopoietins 1 and 2, abolished cell migration induced by MSC-EVs [76].

## Functional proteins of MSC-EVs in other diseases

Cystinosis is a rare disease caused by homozygous mutations of the cystinosin gene, resulting in the accumulation of intralysosomal cystine in the body. MSC-EVs alleviated the accumulation of cystine in skin fibroblasts from cystinosis patients in vitro. MSC-EVs also transferred wild-type cystinosin to the skin fibroblasts [77]. Mao et al. reported that ubiquitin protein ligase E3 component n-recognin 2 (UBR2) was enriched in MSC-EVs with p53 deficiency. MSC-EVs with p53 deficiency could be internalized into murine foregastric carcinoma cells, resulting in the overexpression of UBR2. Additionally, UBR2 activated the Wnt/β-catenin signaling pathway to promote the growth and metastasis of gastric cancer [78].

## Conclusions

It has been well established that MSC-EVs have the same immunomodulatory and anti-inflammatory effects as their parental cells. Compared with MSCs, MSC-EVs offer several advantages including lower immunogenicity and an improved safety profile. Therefore, MSC-EVs may serve as an alternative to whole*-*cell therapy for the treatment of a myriad of diseases. The therapeutic effects of MSC-EVs are at least partially mediated by delivering functional proteins to the recipient cells. MSC-EVs obtained from different origins and preparation methodologies have similar beneficial effects, indicating similar protein compositions of MSC-EVs. Proteomic analysis in MSC-EVs has identified hundreds of proteins in key biological processes such as EV biogenesis, cellular structure, tissue repair and regeneration, and inflammatory response. Approximately 40 of the functional proteins responsible for the protective effects of MSC-EVs are listed in the present review. However, research in functional proteins of MSC-EVs faces several challenges. Foremost, the sorting mechanisms of proteins into MSC-EVs are not clear. The existing sorting pathways apply mainly to proteins with post-translational modifications. Secondly, there is a lack of methodology to efficiently target MSC-EVs to specific tissue types, although targeting attempts have been made by altering EVs via chemical modification and manipulating parental cells via genetic modification. Lastly, it is unknown whether functional miRNAs and proteins work independently or synergistically, giving that miRNAs are also transferred from MSC-EVs to target cells. Further studies are warranted to evaluate MSC-EV therapeutics based on the delivery of functional proteins.

## Data Availability

Not applicable.

## Abbreviations

AV: Annexin-V
BDNF: Brain-derived neurotrophic factor
BM: Bone marrow
CCR2: C-C motif chemokine receptor-2
CTB: Cholera toxin B
EMMPRIN: Extracellular matrix metalloproteinase inducer
EP4: Prostaglandin E_2_ receptor 4
ESCRT: Endosomal sorting complex required for transport
EVs: Extracellular vesicles
HGF: Hepatocyte growth factor
HIF1-α: Hypoxia-inducible factor 1-alpha
ISEV: International Society for Extracellular Vesicles
miRNA: MicroRNA
MSC-EVs: MSC-derived EVs
MSCs: Mesenchymal stem (stromal) cells
MVB: Multivesicular bodies
PDGF-D: Platelet-derived growth factor D
PD-L1: Programmed death ligand-1
RISC: RNA-induced silencing complex
SCF: Stem cell factor
SNAREs: Soluble *N*-ethylmaleimide-sensitive factor attachment protein receptors
ST: Shiga toxin
TGF-β: Transforming growth factor β
UBR2: Ubiquitin protein ligase E3 component n-recognin 2
VEGF: Vascular endothelial growth factor

## References

[1] Zheng G, Huang L, Tong H, Shu Q, Hu Y, Ge M, Deng K, Zhang L, Zou B, Cheng B, Xu J (2014). Treatment of acute respiratory distress syndrome with allogeneic adipose-derived mesenchymal stem cells: a randomized, placebo-controlled pilot study. Respir Res.

[2] Florea V, Rieger AC, DiFede DL, El-Khorazaty J, Natsumeda M, Banerjee MN, Tompkins BA, Khan A, Schulman IH, Landin AM (2017). Dose comparison study of allogeneic mesenchymal stem cells in patients with ischemic cardiomyopathy (the TRIDENT study). Circ Res.

[3] Smets Françoise, Dobbelaere Dries, McKiernan Patrick, Dionisi-Vici Carlo, Broué Pierre, Jacquemin Emmanuel, Lopes Ana Isabel, Gonçalves Isabel, Mandel Hanna, Pawlowska Joanna, Kamińska Diana, Shteyer Eyal, Torre Giuliano, Shapiro Riki, Eyskens François, Clapuyt Philippe, Gissen Paul, Pariente Danièle, Grunewald Stephanie, Yudkoff Marc, Binda Maria Mercedes, Najimi Mustapha, Belmonte Nathalie, de Vos Beatrice, Thonnard Joelle, Sokal Etienne (2019). Phase I/II Trial of Liver–derived Mesenchymal Stem Cells in Pediatric Liver–based Metabolic Disorders. Transplantation.

[4] Thery C, Witwer KW, Aikawa E, Alcaraz MJ, Anderson JD, Andriantsitohaina R, Antoniou A, Arab T, Archer F, Atkin-Smith GK (2018). Minimal information for studies of extracellular vesicles 2018 (MISEV2018): a position statement of the International Society for Extracellular Vesicles and update of the MISEV2014 guidelines. J Extracell Vesicles.

[5] Hessvik NP, Llorente A (2018). Current knowledge on exosome biogenesis and release. Cell Mol Life Sci.

[6] Meehan B, Rak J, Di Vizio D (2016). Oncosomes - large and small: what are they, where they came from?. J Extracell Vesicles.

[7] Holm Mea M., Kaiser Julia, Schwab Martin E. (2018). Extracellular Vesicles: Multimodal Envoys in Neural Maintenance and Repair. Trends in Neurosciences.

[8] Kowal J, Arras G, Colombo M, Jouve M, Morath JP, Primdal-Bengtson B, Dingli F, Loew D, Tkach M, Thery C (2016). Proteomic comparison defines novel markers to characterize heterogeneous populations of extracellular vesicle subtypes. Proc Natl Acad Sci U S A.

[9] Ramos TL, Sanchez-Abarca LI, Muntion S, Preciado S, Puig N, Lopez-Ruano G, Hernandez-Hernandez A, Redondo A, Ortega R, Rodriguez C (2016). MSC surface markers (CD44, CD73, and CD90) can identify human MSC-derived extracellular vesicles by conventional flow cytometry. Cell Commun Signal.

[10] Robb KP, Fitzgerald JC, Barry F, Viswanathan S (2019). Mesenchymal stromal cell therapy: progress in manufacturing and assessments of potency. Cytotherapy.

[11] Kordelas L, Rebmann V, Ludwig AK, Radtke S, Ruesing J, Doeppner TR, Epple M, Horn PA, Beelen DW, Giebel B (2014). MSC-derived exosomes: a novel tool to treat therapy-refractory graft-versus-host disease. Leukemia.

[12] Linares R, Tan S, Gounou C, Arraud N, Brisson AR (2015). High-speed centrifugation induces aggregation of extracellular vesicles. J Extracell Vesicles.

[13] Webber Jason, Clayton Aled (2013). How pure are your vesicles?. Journal of Extracellular Vesicles.

[14] Tomasoni S, Longaretti L, Rota C, Morigi M, Conti S, Gotti E, Capelli C, Introna M, Remuzzi G, Benigni A (2013). Transfer of growth factor receptor mRNA via exosomes unravels the regenerative effect of mesenchymal stem cells. Stem Cells Dev.

[15] Tang XD, Shi L, Monsel A, Li XY, Zhu HL, Zhu YG, Qu JM (2017). Mesenchymal stem cell microvesicles attenuate acute lung injury in mice partly mediated by Ang-1 mRNA. Stem Cells.

[16] Collino F, Bruno S, Incarnato D, Dettori D, Neri F, Provero P, Pomatto M, Oliviero S, Tetta C, Quesenberry PJ, Camussi G (2015). AKI recovery induced by mesenchymal stromal cell-derived extracellular vesicles carrying microRNAs. J Am Soc Nephrol.

[17] Wang X, Gu H, Qin D, Yang L, Huang W, Essandoh K, Wang Y, Caldwell CC, Peng T, Zingarelli B, Fan GC (2015). Exosomal miR-223 contributes to mesenchymal stem cell-elicited cardioprotection in polymicrobial sepsis. Sci Rep.

[18] Chen L, Lu FB, Chen DZ, Wu JL, Hu ED, Xu LM, Zheng MH, Li H, Huang Y, Jin XY (2018). BMSCs-derived miR-223-containing exosomes contribute to liver protection in experimental autoimmune hepatitis. Mol Immunol.

[19] Xin H, Katakowski M, Wang F, Qian JY, Liu XS, Ali MM, Buller B, Zhang ZG, Chopp M (2017). MicroRNA cluster miR-17-92 cluster in exosomes enhance neuroplasticity and functional recovery after stroke in rats. Stroke.

[20] Colombo M, Moita C, van Niel G, Kowal J, Vigneron J, Benaroch P, Manel N, Moita LF, Thery C, Raposo G (2013). Analysis of ESCRT functions in exosome biogenesis, composition and secretion highlights the heterogeneity of extracellular vesicles. J Cell Sci.

[21] Hoshino D, Kirkbride KC, Costello K, Clark ES, Sinha S, Grega-Larson N, Tyska MJ, Weaver AM (2013). Exosome secretion is enhanced by invadopodia and drives invasive behavior. Cell Rep.

[22] Stuffers S, Sem Wegner C, Stenmark H, Brech A (2009). Multivesicular endosome biogenesis in the absence of ESCRTs. Traffic.

[23] Chairoungdua A, Smith DL, Pochard P, Hull M, Caplan MJ (2010). Exosome release of beta-catenin: a novel mechanism that antagonizes Wnt signaling. J Cell Biol.

[24] Hurwitz SN, Conlon MM, Rider MA, Brownstein NC, Meckes DG (2016). Nanoparticle analysis sheds budding insights into genetic drivers of extracellular vesicle biogenesis. J Extracell Vesicles.

[25] Hurwitz SN, Nkosi D, Conlon MM, York SB, Liu X, Tremblay DC, Meckes DG Jr. CD63 regulates Epstein-Barr virus LMP1 exosomal packaging, enhancement of vesicle production, and noncanonical NF-kappaB signaling. J Virol. 2017;91:e02251–16. 10.1128/JVI.02251-16 PMC5309960 27974566

[26] Hurley JH (2015). ESCRTs are everywhere. EMBO J.

[27] Dores MR, Chen B, Lin H, Soh UJ, Paing MM, Montagne WA, Meerloo T, Trejo J (2012). ALIX binds a YPX(3)L motif of the GPCR PAR1 and mediates ubiquitin-independent ESCRT-III/MVB sorting. J Cell Biol.

[28] Dores MR, Grimsey NJ, Mendez F, Trejo J (2016). ALIX regulates the ubiquitin-independent lysosomal sorting of the P2Y1 purinergic receptor via a YPX3L motif. PLoS One.

[29] Yamashita Y, Kojima K, Tsukahara T, Agawa H, Yamada K, Amano Y, Kurotori N, Tanaka N, Sugamura K, Takeshita T (2008). Ubiquitin-independent binding of Hrs mediates endosomal sorting of the interleukin-2 receptor beta-chain. J Cell Sci.

[30] van Niel G, Charrin S, Simoes S, Romao M, Rochin L, Saftig P, Marks MS, Rubinstein E, Raposo G (2011). The tetraspanin CD63 regulates ESCRT-independent and -dependent endosomal sorting during melanogenesis. Dev Cell.

[31] McKenzie AJ, Hoshino D, Hong NH, Cha DJ, Franklin JL, Coffey RJ, Patton JG, Weaver AM (2016). KRAS-MEK signaling controls Ago2 sorting into exosomes. Cell Rep.

[32] Itoh S, Mizuno K, Aikawa M, Aikawa E (2018). Dimerization of sortilin regulates its trafficking to extracellular vesicles. J Biol Chem.

[33] Baker RW, Hughson FM (2016). Chaperoning SNARE assembly and disassembly. Nat Rev Mol Cell Biol.

[34] Savina A, Vidal M, Colombo MI (2002). The exosome pathway in K562 cells is regulated by Rab11. J Cell Sci.

[35] Jae N, McEwan DG, Manavski Y, Boon RA, Dimmeler S (2015). Rab7a and Rab27b control secretion of endothelial microRNA through extracellular vesicles. FEBS Lett.

[36] Hsu C, Morohashi Y, Yoshimura S, Manrique-Hoyos N, Jung S, Lauterbach MA, Bakhti M, Gronborg M, Mobius W, Rhee J (2010). Regulation of exosome secretion by Rab35 and its GTPase-activating proteins TBC1D10A-C. J Cell Biol.

[37] Ostrowski M, Carmo NB, Krumeich S, Fanget I, Raposo G, Savina A, Moita CF, Schauer K, Hume AN, Freitas RP (2010). Rab27a and Rab27b control different steps of the exosome secretion pathway. Nat Cell Biol.

[38] Hoshino A, Costa-Silva B, Shen TL, Rodrigues G, Hashimoto A, Tesic Mark M, Molina H, Kohsaka S, Di Giannatale A, Ceder S (2015). Tumour exosome integrins determine organotropic metastasis. Nature.

[39] Nazarenko I, Rana S, Baumann A, McAlear J, Hellwig A, Trendelenburg M, Lochnit G, Preissner KT, Zoller M (2010). Cell surface tetraspanin Tspan8 contributes to molecular pathways of exosome-induced endothelial cell activation. Cancer Res.

[40] Segura E, Nicco C, Lombard B, Veron P, Raposo G, Batteux F, Amigorena S, Thery C (2005). ICAM-1 on exosomes from mature dendritic cells is critical for efficient naive T-cell priming. Blood.

[41] Bonjoch L, Gironella M, Iovanna JL, Closa D (2017). REG3beta modifies cell tumor function by impairing extracellular vesicle uptake. Sci Rep.

[42] Kim HS, Choi DY, Yun SJ, Choi SM, Kang JW, Jung JW, Hwang D, Kim KP, Kim DW (2012). Proteomic analysis of microvesicles derived from human mesenchymal stem cells. J Proteome Res.

[43] Angulski AB, Capriglione LG, Batista M, Marcon BH, Senegaglia AC, Stimamiglio MA, Correa A (2017). The protein content of extracellular vesicles derived from expanded human umbilical cord blood-derived CD133(+) and human bone marrow-derived mesenchymal stem cells partially explains why both sources are advantageous for regenerative medicine. Stem Cell Rev.

[44] Anderson JD, Johansson HJ, Graham CS, Vesterlund M, Pham MT, Bramlett CS, Montgomery EN, Mellema MS, Bardini RL, Contreras Z (2016). Comprehensive proteomic analysis of mesenchymal stem cell exosomes reveals modulation of angiogenesis via nuclear factor-KappaB signaling. Stem Cells.

[45] La Greca A, Solari C, Furmento V, Lombardi A, Biani MC, Aban C, Moro L, Garcia M, Guberman AS, Sevlever GE (2018). Extracellular vesicles from pluripotent stem cell-derived mesenchymal stem cells acquire a stromal modulatory proteomic pattern during differentiation. Exp Mol Med.

[46] Lai RC, Tan SS, Teh BJ, Sze SK, Arslan F, de Kleijn DP, Choo A, Lim SK (2012). Proteolytic potential of the MSC exosome proteome: implications for an exosome-mediated delivery of therapeutic proteasome. Int J Proteomics.

[47] Lai RC, Tan SS, Yeo RW, Choo AB, Reiner AT, Su Y, Shen Y, Fu Z, Alexander L, Sze SK, Lim SK (2016). MSC secretes at least 3 EV types each with a unique permutation of membrane lipid, protein and RNA. J Extracell Vesicles.

[48] Otero-Ortega L, Laso-Garcia F, Gomez-de Frutos MD, Rodriguez-Frutos B, Pascual-Guerra J, Fuentes B, Diez-Tejedor E, Gutierrez-Fernandez M (2017). White matter repair after extracellular vesicles administration in an experimental animal model of subcortical stroke. Sci Rep.

[49] Eirin A, Zhu XY, Puranik AS, Woollard JR, Tang H, Dasari S, Lerman A, van Wijnen AJ, Lerman LO (2016). Comparative proteomic analysis of extracellular vesicles isolated from porcine adipose tissue-derived mesenchymal stem/stromal cells. Sci Rep.

[50] Eirin A, Zhu XY, Puranik AS, Woollard JR, Tang H, Dasari S, Lerman A, van Wijnen AJ, Lerman LO (2017). Integrated transcriptomic and proteomic analysis of the molecular cargo of extracellular vesicles derived from porcine adipose tissue-derived mesenchymal stem cells. PLoS One.

[51] Eirin Alfonso, Zhu Xiang‐Yang, Woollard John R., Tang Hui, Dasari Surendra, Lerman Amir, Lerman Lilach O. (2019). Metabolic Syndrome Interferes with Packaging of Proteins within Porcine Mesenchymal Stem Cell‐Derived Extracellular Vesicles. STEM CELLS Translational Medicine.

[52] van Balkom BWM, Gremmels H, Giebel B, Lim SK (2019). Proteomic signature of mesenchymal stromal cell-derived small extracellular vesicles. Proteomics.

[53] Mokarizadeh A, Delirezh N, Morshedi A, Mosayebi G, Farshid AA, Mardani K (2012). Microvesicles derived from mesenchymal stem cells: potent organelles for induction of tolerogenic signaling. Immunol Lett.

[54] Crain SK, Robinson SR, Thane KE, Davis AM, Meola DM, Barton BA, Yang VK, Hoffman AM (2019). Extracellular vesicles from Wharton’s jelly mesenchymal stem cells suppress CD4 expressing T cells through transforming growth factor beta and adenosine signaling in a canine model. Stem Cells Dev.

[55] Alvarez V, Sanchez-Margallo FM, Macias-Garcia B, Gomez-Serrano M, Jorge I, Vazquez J, Blazquez R, Casado JG (2018). The immunomodulatory activity of extracellular vesicles derived from endometrial mesenchymal stem cells on CD4+ T cells is partially mediated by TGFbeta. J Tissue Eng Regen Med.

[56] Adamo A, Brandi J, Caligola S, Delfino P, Bazzoni R, Carusone R, Cecconi D, Giugno R, Manfredi M, Robotti E (2019). Extracellular vesicles mediate mesenchymal stromal cell-dependent regulation of B cell PI3K-AKT signaling pathway and actin cytoskeleton. Front Immunol.

[57] Harting MT, Srivastava AK, Zhaorigetu S, Bair H, Prabhakara KS, Toledano Furman NE, Vykoukal JV, Ruppert KA, Cox CS, Olson SD (2018). Inflammation-stimulated mesenchymal stromal cell-derived extracellular vesicles attenuate inflammation. Stem Cells.

[58] Chen SY, Lin MC, Tsai JS, He PL, Luo WT, Herschman H, Li HJ. EP4 antagonist-elicited extracellular vesicles from mesenchymal stem cells rescue cognition/learning deficiencies by restoring brain cellular functions. Stem Cells Transl Med. 2019;8:707–23. 10.1002/sctm.18-0284 PMC6591556 30891948

[59] Katsuda T, Tsuchiya R, Kosaka N, Yoshioka Y, Takagaki K, Oki K, Takeshita F, Sakai Y, Kuroda M, Ochiya T (2013). Human adipose tissue-derived mesenchymal stem cells secrete functional neprilysin-bound exosomes. Sci Rep.

[60] de Godoy MA, Saraiva LM, de Carvalho LRP, Vasconcelos-Dos-Santos A, Beiral HJV, Ramos AB, Silva LRP, Leal RB, Monteiro VHS, Braga CV (2018). Mesenchymal stem cells and cell-derived extracellular vesicles protect hippocampal neurons from oxidative stress and synapse damage induced by amyloid-beta oligomers. J Biol Chem.

[61] McBride JD, Rodriguez-Menocal L, Candanedo A, Guzman W, Garcia-Contreras M, Badiavas EV (2018). Dual mechanism of type VII collagen transfer by bone marrow mesenchymal stem cell extracellular vesicles to recessive dystrophic epidermolysis bullosa fibroblasts. Biochimie.

[62] Zhang B, Wang M, Gong A, Zhang X, Wu X, Zhu Y, Shi H, Wu L, Zhu W, Qian H, Xu W (2015). HucMSC-exosome mediated-Wnt4 signaling is required for cutaneous wound healing. Stem Cells.

[63] Zhang B, Shi Y, Gong A, Pan Z, Shi H, Yang H, Fu H, Yan Y, Zhang X, Wang M (2016). HucMSC exosome-delivered 14-3-3zeta orchestrates self-control of the Wnt response via modulation of YAP during cutaneous regeneration. Stem Cells.

[64] Shabbir A, Cox A, Rodriguez-Menocal L, Salgado M, Van Badiavas E (2015). Mesenchymal stem cell exosomes induce proliferation and migration of normal and chronic wound fibroblasts, and enhance angiogenesis in vitro. Stem Cells Dev.

[65] Ahn SY, Park WS, Kim YE, Sung DK, Sung SI, Ahn JY, Chang YS (2018). Vascular endothelial growth factor mediates the therapeutic efficacy of mesenchymal stem cell-derived extracellular vesicles against neonatal hyperoxic lung injury. Exp Mol Med.

[66] Wang H, Zheng R, Chen Q, Shao J, Yu J, Hu S (2017). Mesenchymal stem cells microvesicles stabilize endothelial barrier function partly mediated by hepatocyte growth factor (HGF). Stem Cell Res Ther.

[67] Gennai S, Monsel A, Hao Q, Park J, Matthay MA, Lee JW (2015). Microvesicles derived from human mesenchymal stem cells restore alveolar fluid clearance in human lungs rejected for transplantation. Am J Transplant.

[68] Hu S, Park J, Liu A, Lee J, Zhang X, Hao Q, Lee JW (2018). Mesenchymal stem cell microvesicles restore protein permeability across primary cultures of injured human lung microvascular endothelial cells. Stem Cells Transl Med.

[69] Eirin A, Zhu XY, Puranik AS, Tang H, McGurren KA, van Wijnen AJ, Lerman A, Lerman LO (2017). Mesenchymal stem cell-derived extracellular vesicles attenuate kidney inflammation. Kidney Int.

[70] Shen B, Liu J, Zhang F, Wang Y, Qin Y, Zhou Z, Qiu J, Fan Y (2016). CCR2 positive exosome released by mesenchymal stem cells suppresses macrophage functions and alleviates ischemia/reperfusion-induced renal injury. Stem Cells Int.

[71] Jiang ZZ, Liu YM, Niu X, Yin JY, Hu B, Guo SC, Fan Y, Wang Y, Wang NS (2016). Exosomes secreted by human urine-derived stem cells could prevent kidney complications from type I diabetes in rats. Stem Cell Res Ther.

[72] Ma J, Zhao Y, Sun L, Sun X, Zhao X, Sun X, Qian H, Xu W, Zhu W (2017). Exosomes derived from Akt-modified human umbilical cord mesenchymal stem cells improve cardiac regeneration and promote angiogenesis via activating platelet-derived growth factor D. Stem Cells Transl Med.

[73] Gonzalez-King H, Garcia NA, Ontoria-Oviedo I, Ciria M, Montero JA, Sepulveda P (2017). Hypoxia inducible factor-1alpha potentiates jagged 1-mediated angiogenesis by mesenchymal stem cell-derived exosomes. Stem Cells.

[74] Vrijsen KR, Maring JA, Chamuleau SA, Verhage V, Mol EA, Deddens JC, Metz CH, Lodder K, van Eeuwijk EC, van Dommelen SM (2016). Exosomes from cardiomyocyte progenitor cells and mesenchymal stem cells stimulate angiogenesis via EMMPRIN. Adv Healthc Mater.

[75] Lopatina T, Bruno S, Tetta C, Kalinina N, Porta M, Camussi G (2014). Platelet-derived growth factor regulates the secretion of extracellular vesicles by adipose mesenchymal stem cells and enhances their angiogenic potential. Cell Commun Signal.

[76] Wysoczynski M, Pathan A, Moore JB, Farid T, Kim J, Nasr M, Kang Y, Li H, Bolli R (2019). Pro-angiogenic actions of CMC-derived extracellular vesicles rely on selective packaging of angiopoietin 1 and 2, but not FGF-2 and VEGF. Stem Cell Rev.

[77] Iglesias DM, El-Kares R, Taranta A, Bellomo F, Emma F, Besouw M, Levtchenko E, Toelen J, van den Heuvel L, Chu L (2012). Stem cell microvesicles transfer cystinosin to human cystinotic cells and reduce cystine accumulation in vitro. PLoS One.

[78] Mao J, Liang Z, Zhang B, Yang H, Li X, Fu H, Zhang X, Yan Y, Xu W, Qian H (2017). UBR2 enriched in p53 deficient mouse bone marrow mesenchymal stem cell-exosome promoted gastric cancer progression via Wnt/beta-catenin pathway. Stem Cells.

[79] Di Trapani M, Bassi G, Midolo M, Gatti A, Kamga PT, Cassaro A, Carusone R, Adamo A, Krampera M (2016). Differential and transferable modulatory effects of mesenchymal stromal cell-derived extracellular vesicles on T, B and NK cell functions. Sci Rep.

[80] El-Darouti M, Fawzy M, Amin I, Abdel Hay R, Hegazy R, Gabr H, El Maadawi Z (2016). Treatment of dystrophic epidermolysis bullosa with bone marrow non-hematopoeitic stem cells: a randomized controlled trial. Dermatol Ther.

